# Spatial differentiation and driving factors of the high-quality development of undertakings for the aged of China

**DOI:** 10.1186/s12939-023-01921-7

**Published:** 2023-05-26

**Authors:** Rong Peng, Jianhang Huang, Xueqin Deng, Yingying Wang

**Affiliations:** Institute of New Development, Guangdong University of Finance and Economics, Guangzhou, China

**Keywords:** High-quality development, Undertakings for the aged, Spatial differentiation, Spatial panel regression

## Abstract

**Background:**

Promoting the high-quality development (HQD) of undertakings for the aged is an important action to proactively respond to the rapid population aging in China. This study analyzes spatial differentiation and driving factors of the HQD of China’s undertakings for the aged.

**Methods:**

Based on a quantitative indicator system consisting of old-age social security, elder care services, health care service and older adults’ social participation, the HQD levels of 31 Chinese provincial administrative regions during 2013–2019 are measured by using the entropy weight method. Spatial panel regression models are used to analyze the impact of population aging, economic development and digital technology on the HQD of undertakings for the aged.

**Results:**

The comprehensive level of the HQD slightly increased from 0.212 to 2013 to 0.220 in 2019, and the overall level was low. The HQD of the eastern region was the highest (0.292), followed by the western region (0.215), and the central region was the lowest (0.151). The high-high cluster type was mainly distributed in the eastern region; the low-low cluster type was mainly distributed in the western and central regions. Economic development and digital technology have significant positive effects while population aging has significant negative effects on the HQD of undertakings for the aged.

**Conclusion:**

There is a significantly spatial differentiation in the HQD of China’s undertakings for the aged. In order to promote the HQD of undertakings for the aged, it is necessary to identify development gaps through making HQD evaluation and to focus on the indicators that are critical in maintaining sustainable economic development and to develop digital technology in order to get rid of those gaps.

**Supplementary Information:**

The online version contains supplementary material available at 10.1186/s12939-023-01921-7.

## Introduction

Developing the government-led old-age programs is a common action in the global society to address the challenges of increasing aging problem [1–3]. The undertakings for the aged aim to ensure basic public service, the sustainable social security and pension system, the healthcare system and the long-term care system [4–6]. In China, there are 267.4 million people aged 60 or over, accounting for 18.9% of the total population [7]. To response to the great challenges of population aging, the Chinese central government put forward the “14th Five-Year Plan” for developing the undertakings for the aged and set up the main goal of promoting the high-quality development (HQD) of undertakings for the aged [6]. The concept of HQD was first proposed at the 19th National Congress of the Communist Party of China in 2017, indicating that China has changed from the development mode of pursuing high-speed economic growth to the mode of pursuing quality first and fostering new growth drivers [8]. From the perspectives of quantity and quality, promoting the HQD of undertakings for the aged means that China will take further steps to develop more high-quality public programs rather than achieve an increase in the number of old-age programs.

During the last decade, China’s undertakings for the aged had gone through a rapid development. The prototype of the old-age welfare policy framework had begun to take shape, and the elder care system had been greatly improved. For instance, the number of elder care beds per 1000 older adults increased from 17.7 to 2010 to 32.7 in 2020. The basic old-age insurance system covered 1028.7 million people in 2021, accounting for 72.9% of the population [7]. However, the overall level of undertakings for the aged development in China is not high. The basic old-age pension benefits for the elderly people were at relatively low level, with only 179 Yuan per capita every month [7]. The social care service system for the old people is underdeveloped and can’t meet their needs for social support [9, 10]. The community care service for older adults is not only insufficient in quantity but also of low quality [9]. Only 7% of older adults relied on government or market social care services when they need help [10]. In addition, the undertakings for the aged vary greatly between urban and rural areas. The development of old-age program in rural area is lagging far behind that of urban area [11–13]. Therefore, it is extremely important for Chinese government to promote the quality of undertakings for the aged in China.

The research of HQD mainly focuses on the following four aspects: the scientific connotation, the evaluation index system, the measurement and the influencing factors of HDQ. The connotation of HDQ is usually explained from two perspectives, one of which is the five development concepts of innovation, coordination, green, openness, and shared development [8, 14, 15], the other is the coupling development of society-economy-ecology system including high-quality economic development, high-quality social development and high-quality environmental development [16–18]. For instance, Pan et al. (2021) interpreted the connotation of HQD from five dimensions: economic development, innovation efficiency, environmental impact, ecological services, and people’s livelihood [8]. Some studies measured the comprehensive development level of HQD at the national level [8, 14, 15, 19, 20]. Others measured the HQD in sectors such as agriculture [21], manufacturing [22], marine fishery [23], tourism [24], the HQD of urban and urbanization [25, 26], and urban agglomerations [27]. In recent years, sustainable spatial development had attracted attention of many researchers and the spatial development model of HQD had become a focus of research [17, 19, 20, 27]. The researchers mainly examined the impact of digital economy [17], technological innovation [19], environmental regulation [28] and foreign direct investment [29] on high-quality development. For instance, Ding et al. (2022) examined the impact of the digital economy on the level of high-quality economic development in 30 Chinese provinces by using a mediating effects model and a spatial Durbin model [19]. Due to the different understandings of the connotation of HQD and the perspective of multidimensional measurement, the evaluation results of the literature on HQD often lack comparability.

However, there are few literatures on the HQD of undertakings for the aged in China. Although several researches have studied the current situation and problems of the development of old-age programs [30, 31], the connotation of the HQD of China’s undertakings for the aged have remained unexplored in depth, and there is a lack of measurement and evaluation of HQD of undertakings for the aged. To our knowledge, there is no research on the spatial characteristics and evolution trend of the HQD of China’s undertakings for the aged.

As the largest emerging economy with the largest aging population in the world, China’s action strategy to population aging proposed some interesting questions worth exploring. For example, during the specific transition stage from high-speed to high-quality development, has China improved its undertakings for the aged while improving the quality of economy? What are the main factors influencing the HQD of undertakings for the aged in China? What does China’s HQD of undertakings for the aged bring to other emerging market countries with aging population? In view of the profound connotation of HQD, it is extremely important to construct measurement index and evaluation model suitable for China’s development characteristics in accordance with China’s actual situation.

This study aims to explore the spatial pattern and driving factors of the HQD of undertakings for the aged in China. Specifically, we construct a comprehensive evaluation index to measure the HQD of undertakings for the aged in 31 Chinese provinces from 2013 to 2019. Furthermore, we investigate the regional differences of HQD from a spatial perspective. In addition, this study analyzes the impact of population aging, economic development and digital technology on the HQD of undertakings for the aged.

The marginal contribution of this study includes at least three aspects. Firstly, it improves a theoretical basis for the connotation of the HQD of undertakings for the aged. Secondly, this study first constructs the measurement indicator system for the HQD of undertakings for the aged and provides academic understanding of the spatial characteristics and evolution trends of the HQD of undertakings for the aged in China, the largest emerging economy in the world. Thirdly, this research endeavors to explore the driving factors of the HQD of undertakings for the aged from the perspective of spatial effects, which provides evidence-based support for the government to formulate policies in order to promote the development of undertakings for the aged.

## Theoretical analysis

### The connotation of HQD of undertakings for the aged

With reference to the research on the connotation of HQD in the literature and on the basis of the main fields of the undertaking for the aged, this study defines the connotation of HQD of the undertaking for the aged from the perspectives of old-age social security, elder care service, elder health service, and social participation of the older adults. First, the HQD of undertakings for the aged means a more comprehensive social security system for the elderly people. The basic goal of the HQD of undertakings for the aged is to establish a more equitable and sustainable social security system for the geriatric society [6]. Currently, China’s social security system is subject to the level of economic development, and there is an imbalance in the development of old-age program that the rural areas significantly lag behind the urban areas [11–13]. The HQD of undertakings for the aged is to establish a social security system for the elderly people that adapts to the economic development, and to formulate financial supporting policies for the old people. In terms of measurement dimensions, the high-quality undertakings for the aged should include not only basic social security such as basic social endowment insurance and basic social medical insurance, but also old age subsidy system, care subsidy system and other financial subsidy systems specially designed for the elderly people.

Second, the HQD of undertakings for the aged is manifested as a high-quality elder care system. High-quality elder care system has a strong social care service network and provides a variety of care services such as daily life assistance, community engagement support services, psychological comfort services and other support services. The HQD of undertakings for the aged requires that the elder care service facilities cover nationwide areas and benefit all the population; there is a continuous increase in the supply of care services and the gap between different regions narrows; the quantity and quality of workforce resources to provide elder care services have been improved to meet the older adults’ needs [32].

Third, the HQD of undertakings for the aged is manifested as a high-quality health support system for the older adults. High-quality development indicates the continuous improvement of public health and medical service systems, the increasing supply of elderly health services resources, the improvement of the allocation of elderly health research talents, and the availability of health support systems [33]. At the same time, the health support system could be accessed more easily through improving the capacity of community-based medical services and providing home medical services such as family care beds and home visits. The ultimate goal of the HQD of undertakings for the aged is to better meet the health needs of the elderly people and to improve the health level of them.

Another aspect of the HQD of undertakings for the aged is the improvement in the elderly people’s ability and level of social participation [6]. The HQD of undertakings for the aged attaches importance to the positive aging orientation of “rights-based”, encouraging the elderly people to participate in social activities to optimize the health attainment of the elderly [34]. From an international perspective, the theory of geriatric society has roughly undergone an evolution from “successful aging” to “healthy aging” and to “active aging” [3]. Different from “successful aging” and “healthy aging”, the theory of “active aging” not only emphasizes the health status of physiological, psychological and social functions, but also changes the focus of strategic planning from “demand-based” to “rights-based” [3], thereby optimizing the access to health, participation and security of the elderly, which has become a new development strategy for the international community to deal with the problem of aging [2]. Therefore, the higher the degree or level of social engagement of the elderly, the more likely it is to promote the social integration, mental health and physical health of the elderly, representing a higher level of development in the cause of aging.

### Driving forces of HQD of undertakings for the aged and hypothesis

Based on the connotation of the HQD of undertakings for the aged, it is mainly affected by population aging, economic development, technological innovation, and policy force. The population aging determines the demand for elder care service and financial sources of the undertaking for the aged, which affects the HQD on the demand side. Economic development provides support of financial sources and services, which affects the HQD on the supply side. The development of digital technology is the technological innovation that affects the efficiency on the supply side of the HQD. The policy is the external driving force that affects the HQD of undertakings for the aged.

#### Population aging

Considering the potential impact of population aging on sustainable development, China implemented a national strategy to actively respond to the aging of the population during the 14th Five-Year Plan period (2021–2025) [35]. The ideas and tasks of the strategy include further improving the social security system, promoting the construction of the social elderly care service system, and providing opportunities and conditions for the elderly people to participate in social life and social development [35]. These tasks obviously overlap the connotation of the HQD of undertakings for the aged. Therefore, population aging is one of the main driving forces to promote the HQD of the undertakings for the aged. Population aging may result in a lower level of HQD with a constant supply capacity. The first hypothesis of this study is proposed based on this.

##### Hypothesis 1

Population aging has a negative impact on the HQD of the undertakings for the aged.

#### Economic development

Economic development is the material basis for promoting people’s well-being [36]. Economic development may not only expand the financial resources for the development of the old-age programs, but also improve the sustainability of social security fund financing, and make it possible to improve social security benefit. In addition, economic development helps to increase public service investment, increase the government-led supply of basic elder care services, and thus improve the development quality of the undertakings for the aged. The higher the level of economic development, the higher the HQD of the undertakings for the aged. The second hypothesis is proposed on the basis of this.

##### Hypothesis 2

Economic development has a positive impact on the HQD of the undertakings for the aged.

#### Digital technology

With the deepening of China’s population aging, China’s labor supply is decreasing, and the economy is increasingly driven by innovation rather than input and investment [37]. In terms of old-age programs, digital technology is conducive to the improvement and exploration of public services [38]. The promotion of digital technology infrastructure such as digital city and 5G application has changed the elder care environment and promoted the participation of the elderly people in society [39, 40]. Hence, digital technology is a new driving force for the HQD of undertakings for the aged. The higher the level of technological innovation, the higher the efficiency of supplying and financing for the elderly care service. Based on this, the third hypothesis is proposed.

##### Hypothesis 3

Digital technology has a positive effect on the HQD of the undertakings for the aged.

#### Policy force

In China, the undertakings for the aged consist of a series of government’s policies and measures to provide the old people with medical care and opportunities of social participation, and a sense of security, worthiness and happiness [6]. China has changed from a stage of rapid growth to a stage of HQD, and the government has proposed to improve the quality of the undertakings for the aged, which means providing more policy support for the older adults. The administrative departments of governments at all levels should increase their investment in the old-age programs and play the role of market supervision, so as to guide investors to provide social elder care services for the elderly. Since policies are mixed with economy and digital technology, which makes it hard to measure, no indicator of policy factor is included in the empirical study.

## Methods

### Data sources and pre-processing

The data are from the 2014–2020 China Civil Affairs Statistics Yearbook, China Statistics Yearbook, China Health Statistics Yearbook, and China Cultural Relics Statistics Yearbook. The data obtained in this study include 31 provincial administrative regions in China. The time span of the data is from 2013 to 2019. The proportion of missing values is less than 4%. For the missing data, the nearest-neighbor interpolation method was used to make up and ensure the continuity of the data. If both the previous and the next data points are available, fill the missing value with the arithmetic mean of the previous and next data points. Otherwise, use the data from the previous or next position to fill in the missing value with the same value. Microsoft Excel 2016 and Stata 17 were used to process and analyze the data. ArcGIS 10.2 was used for the generation of maps.

### Index system for measuring HQD of undertakings for the aged

Based on the connotation of HQD of undertakings for the aged, this study establishes an evaluation index system consisting of four dimensions: old-age social security, elder care services, health care service and elder’s social participation. These four dimensions correspond to the four aspects of the development connotation of HQD of undertakings for the aged. For instance, the dimension of old-age social security corresponds to the first connotation of HQD, a more comprehensive social security system for the elderly people. In consideration of data availability, 19 indicators are included in the evaluation index system with reference to literature [31]. The specific indicators are explained as follows.

The dimension of old-age social security takes fund guarantee into account, mainly involving the social pension insurance expenditure, the public health expenditure and the subsidies for the older adults. The social pension insurance expenditure per capita is used to indicate the level of pension insurance expenditure. Two indicators are included in the public health expenditure: the public health expenditure per capita and social medical insurance expenditure per capita. Three indicators are concerned with the subsidy for the older adults: proportion of population receiving old-age subsidies, care subsidies and pension subsidies.

The dimension of elder care service reflects the facility level and human resources of institutional care and community care, including six specific indicators: number of care beds in communities per thousand older adults, number of community care institutions per thousand older adults, number of institutional care beds per thousand elderly people, number of elder care institutions per thousand elderly people, number of employees in elder care institutions, and number of employees in community care institutions.

The health care service for the elderly people reflects the conditions of providing health support for the elderly people, which could be measured in two aspects: medical facilities and medical care human resources. The number of beds in medical institutions is expressed by the number of beds in geriatric hospital and medical and health institutions per thousand elderly people. Medical care human resources are expressed in terms of the number of health technicians per thousand people and the proportion of registered nurses in health technicians.

Social participation of the elderly people reflects their participation in economic, cultural and social activities through the platform and field of social participation of the elderly people. It is measured by 3 indicators: number of schools for the elderly per thousand people, number of elderly activity centers per thousand people, number of cultural special sessions for the elderly per thousand people. Table 1 lists the formulas and units of all the 19 indicators.

**Table 1:** Index system for evaluating the HQD level of undertakings for the aged

Dimensions	Indicators	Calculation formula	Unit	Weights*
Old-age social security (45.61%) (43.27%)	Social pension insurance expenditure per capita	Basic pension insurance expenditure/number of retired employees	Yuan/person	4.14%
	Public health expenditure per capita	(Government expenditure on health + social expenditure on health)/total population	Yuan/person	7.99%
	Social medical insurance expenditure per capita	Basic medical insurance expenditure/insured population	Yuan/person	6.45%
	Proportion of older adults receiving old-age subsidies	Number of older adults receiving old-age subsidies/population of older adults*100	%	6.02%
	Proportion of older adults receiving elder care subsidies	Number of older adults receiving elder care subsidies/population of older adults*100	%	9.68%
	Proportion of older adults receiving pension subsidies	Number of older adults receiving pension subsidies/population of older adults*100	%	11.34%
Elder care service (25.46%)	Number of institutional care beds per thousand older adults	Number of institutional care beds/population of older adults*1000	Beds/1000 people	3.03%
	Number of elder care institutions per thousand older adults	Number of elder care institutions/population of older adults*1000	Institutions/1000 people	2.94%
	Number of employees in elder care institutions per thousand older adults	Number of employees in elder care institutions/ population of older adults*1000	Employees/1000 people	4.58%
	Number of care beds in communities per thousand older adults	Number of care beds in communities/population of older adults*1000	Beds/1000 people	4.87%
	Number of community care institutions per thousand older adults	Number of community care institutions/population of older adults*1000	Institutions/1000 people	4.50%
	Number of employees in community care institutions per thousand older adults	Number of employees in community care institutions/population of older adults*1000	Employees/1000 people	5.54%
Health care service (10.41%)	Number of beds in geriatric hospital per thousand older adults	Number of beds in geriatric hospitals/population of older adults*1000	Beds/1000 people	4.22%
	Proportion of registered nurses in health technicians	Number of registered nurses/number of health technicians*100	%	0.80%
	Number of health technicians per ten thousand people	Number of health technicians/total population*10,000	Technicians/10,000 people	2.93%
	Number of beds in medical and health institutions per thousand older adults	Number of beds in medical and health institutions/population of older adults*1000	Beds/1000 people	2.46%
Elderly’s social participation (18.52%)	Number of schools for the elderly per thousand people	Number of schools for the elderly/population of older adults*1000	Schools/1000 people	8.83%
	Number of elderly activity centers (rooms and stations) per per thousand people	Number of elderly activity centers/population of older adults*1000	Centers/1000 people	3.87%
	Number of cultural special sessions for the elderly per thousand people	Times/population of older adults*1000	Times/1000 people	5.82%

Note: *The weight is the average of the weights over the years calculated by the entropy weight method

### Method for calculating HQD comprehensive index of undertakings for the aged

The entropy-weight method was used to calculate HQD comprehensive index of undertakings for the aged. The entropy weight method is an objective weighting method, which uses the information entropy to reflect the amount of information obtained for weighting [41]. Information entropy can comprehensively reflect all the information in the sample and the results are of high reliability and robust adaptability. The entropy weight method avoids the interference of subjective factors and ensures that it is more objective and reliable than the subjective method for comprehensive evaluation of multiple indicators [42]. This method has been widely used in the evaluation of sustainable development and high-quality development [42, 43].

For any fixed year from 2013 to 2019, repeat the following steps to calculate the comprehensive development level of HQD of undertakings for the aged. Firstly, the raw data ${Z}_{ij}$ (The subscript i refers to the indicator i (i = 1, 2, ⋯, 18) and j refers to indicator province j ($j=\text{1,2}, \cdots , 31$)) was standardized using the following equation in order to get rid of the influence of dimension and magnitude. For positive values, ${Z}_{ij}^{{\prime }}=\frac{{Z}_{ij}-min{Z}_{ij}}{max{Z}_{ij}-min{Z}_{ij}}$; for negative values, ${Z}_{ij}^{{\prime }}=\frac{{maxZ}_{ij}-{Z}_{ij}}{max{Z}_{ij}-min{Z}_{ij}}$.

Secondly, the information entropy ${E}_{i}$ and the weight ${W}_{i}$ for the indicator i were calculated by the following formulas:

$$\begin{array}{l}{E_i} = - k\sum\limits_j^{31} {{f_{ij}}} {\rm{ln}}\left( {{f_{ij}}} \right)(where,{f_{ij}} = \frac{{Z_{ij}^\prime }}{{\sum\limits_{j = 1}^{31} {Z_{ij}^\prime } }};k = \frac{1}{{ln31}}),\\{\rm{if}}\,{f_{ij}} = 0,{f_{ij}}{\rm{ln}}\left( {{f_{ij}}} \right) = 0.\end{array}$$

$${W}_{i}=\frac{1-{E}_{i}}{18-\sum _{i=1}^{18}{E}_{i}}$$

Thirdly, the weighted index was calculated as follows:

$${Y}_{j}=\sum _{\text{i}}{\text{W}}_{\text{i}}{Z}_{ij}^{{\prime }}$$

The greater the value of ${Y}_{j}$, the higher the level of HQD of the provincial area $j$; on the contrary, the lower the level of the HQD.

### Spatial analysis for HQD of undertakings for the aged

#### Spatial characteristic analysis

To reveal the spatial characteristic in the HQD of undertakings for the aged in China, the software of ArcGIS was used to visualize the comprehensive index. The Natural Breaks Classification (Jenks) was used to classify 31 provincial administrative regions into four categories: Low level area, medium low-level area, medium high-level area and high-level area. The method of Natural Breaks Classification (Jenks) can group the similar values most appropriately to ensure significant differences between groups and small differences within groups [44], is a most widely used classification method for statistical mapping [45].

#### Spatial autocorrelation test

Spatial correlation reflects that the closer the spatial locations of different regions are, the more similar the attributes will be, thus presenting similar spatial phenomena. The First Law of Geography shows that everything has spatial correlation; the closer the distance between the two objects, the greater the spatial correlation [46]. Many studies have found that inter-provincial spatial dependence exists in high-quality development [17, 20, 27, 38]. Spatial autocorrelation test is used to verify whether there is spatial correlation in the HQD of the undertakings for the aged. The global Moran index (Moran’s I) is usually used to test the spatial autocorrelation of variables [47]. Moran I is calculated as follows:

$$\text{M}\text{o}\text{r}\text{a}\text{n}{\prime }\text{s} \text{I}=\frac{\text{N}}{{\text{S}}_{0}}\frac{{\sum }_{\text{i}=1}^{\text{N}}\sum _{\text{j}=1}^{\text{N}}{\text{w}}_{\text{i}\text{j}}({\text{y}}_{\text{i}}-\overline{\text{y}})({\text{y}}_{\text{j}}-\overline{\text{y}})}{{\sum }_{\text{i}=1}^{\text{N}}{({\text{y}}_{\text{i}}-\overline{\text{y}})}^{2}}$$

Where $\text{N}$ denotes the number of space elements, ${y}_{i}$ and ${y}_{j}$ represent the observed value of variable $\text{y}$ in space units i and j, $\overline{y}$ denotes the mean value of the variable y. ${\text{w}}_{\text{i}\text{j}}$ represents the elements in the spatial weight matrix. When constructing the spatial weight matrix, continuous indicators can be used to describe the relationship between spatial objects, such as Euclidean distance, and discrete indicators can also be used to describe the relationship between spatial objects, such as binary distance indicators. The binary distance index takes two values. When the two spatial objects have a common border, the value is 1; Otherwise, the value is zero. Compared with the continuous index, the binary distance index can intuitively represent the adjacency relationship between spatial objects, and the value is 0 or 1, which is easier to understand. On the other hand, the binary adjacency matrix is of high efficiency in calculating, and it can be used and optimized conveniently for matrix operation and graph theory algorithm [48]. This study uses the binary adjacency matrix. ${\text{S}}_{0}$ is the sum of all elements of the space weight matrix.

Global Moran’s I is within the range of [-1, 1]. Positive values represent positive spatial autocorrelation, negative values represent negative spatial autocorrelation, and 0 represents random distribution in space. The larger the absolute value is, the greater the spatial correlation is. When the results of spatial autocorrelation tests show spatial dependence between the observed objects, a spatial measurement model is needed to reflect spatial effect.

Local Moran’s I index reflects whether there is a spatial autocorrelation between a province and neighboring province. The local Moran’s I index is calculated as follows:

$$\text{L}\text{o}\text{c}\text{a}\text{l} \text{M}\text{o}\text{r}\text{a}\text{n}{\prime }\text{s} \text{I}=\frac{{\text{y}}_{\text{i}}-\overline{\text{y}}}{{\text{S}}_{\text{i}}^{2}}\sum _{\text{j}=1,\text{j}\ne \text{i}}^{\text{N}}{\text{w}}_{\text{i}\text{j}}({\text{y}}_{\text{i}}-\overline{\text{y}})$$

where ${S}_{i}^{2}=\frac{\sum _{j=1,j\ne i}^{N}{w}_{ij}}{N-1}-{\overline{y}}^{2}$, Other variables are defined the same as in the global Moran index.

The local Moran’s I index can be shown graphically by Moran’s scatter plot. The Moran’s scatter plot divides the observed values into four categories: the first, second, third and fourth quadrant, corresponding to high-high, low-high, low-low and high-low clustering types of HQD respectively.

#### Spatial panel models

Spatial panel models are linear regression models of panel data combined with spatial lag variables. Compared with the ordinary panel model, the spatial panel model introduces the spatial weight matrix to define the pattern and degree of correlation between the observed units, so as to examine the spatial dependency of the data. The spatial econometric model is more suitable than the ordinary model if the results of the spatial autocorrelation test show that the error terms have spatial autocorrelation [48]. The dependent variable is the HQD of the undertakings for the aged. According to the theoretical analysis in the section of 2.2, the independent variables reflect economic development, population aging and digital technology. Based on the literature and data availability, six indicators were selected as independent variables. With reference to Zeng and Zhao (2019), we use the per capita GDP and the proportion of the tertiary industry as a measure of economic development. Referring to Chen and Liu (2022), we use fixed assets investment and the number of employees in the digital industry as indicators to measure the development of the digital technology [49]. The dependency ratio of the older adults and the proportion of the older adults are commonly used indicators to reflect the aging of the population [50]. The statistical description of the dependent and independent variables is shown in Table 2.

**Table 2:** The statistical description of the dependent and independent variables

Variables	Observed value	Mean	Std.Dev.	Min	Max
Ln(the HQD of the undertakings for the aged)*	217	-1.585	0.428	-2.722	-0.474
The GDP per capita	217	5.762	2.687	2.315	16.422
The proportion of the tertiary industry	217	48.256	9.067	32.000	83.500
The dependency ratio of the older adults	217	14.416	3.403	7.010	23.820
The proportion of the older adults	217	10.407	2.521	4.158	16.131
Ln(the fixed asset investment in digital industries)*	217	4.879	0.987	1.253	6.497
Ln(the number of employees in digital industries)*	217	1.945	1.106	-0.816	4.453

Note: *Ln, natural logarithm

The spatial regression models are used to test the driving factors of HQD of undertakings for the aged. The spatial error model (SEM), the spatial autoregressive (SAR) model, spatial Dubin model (SDM) and spatial autocorrelation (SAC) model are the most wildly used spatial econometric models [51]. The models of SEM (Model 1), SAR (Model 2), SDM (Model 3) and SAC (Model 4) in this study can be expressed as follows:

Model 1 $${y}_{it}=\beta {x}_{it}+\lambda W{\mu }_{it}+{\epsilon }_{it}$$

Model 2 $${y}_{it}=\rho W{y}_{it}+\beta {x}_{it}+{\epsilon }_{it}$$

Model 3 $${y}_{it}=\rho W{y}_{it}+\beta {x}_{it}+\theta W{x}_{it}+{\epsilon }_{it}$$

Model 4 $${y}_{it}=\rho W{y}_{it}+\beta {x}_{it}+\lambda W{u}_{it}+{\epsilon }_{it}$$

Where, ${y}_{it}$ represents the HQD level of the i^th^ province in year t, ${x}_{ij}$ represents the independent variable vector. $\lambda$, $\rho$ and $\theta$ are spatial autoregression parameters. $\beta$ is the regression parameter. $W$ is the binary adjacency matrix. In order to test the stability of the SAC model, we also run a SAC model based on the geographical distance matrix. Both $W{y}_{it}$ and $W{x}_{it}$ are spatially lagged variables. $W{u}_{it}$ is a spatially lagged error term. ${\epsilon }_{it}$ is an error term.

The LM test, LR test and Wald test were used to make comparison among SDM, SEM and SAR models in order to select the most appropriate one of these models. For the comparison between SDM and SAC, we usually use the indicators of AIC, BIC and R-square. Generally speaking, the lower the AIC or BIC value and the higher R-squared value, the better the model is. The Hausman’s test was used to determine fixed effect or random effect.

## Results

### Development level of HQD of undertakings for the aged

**Fig. 1:**
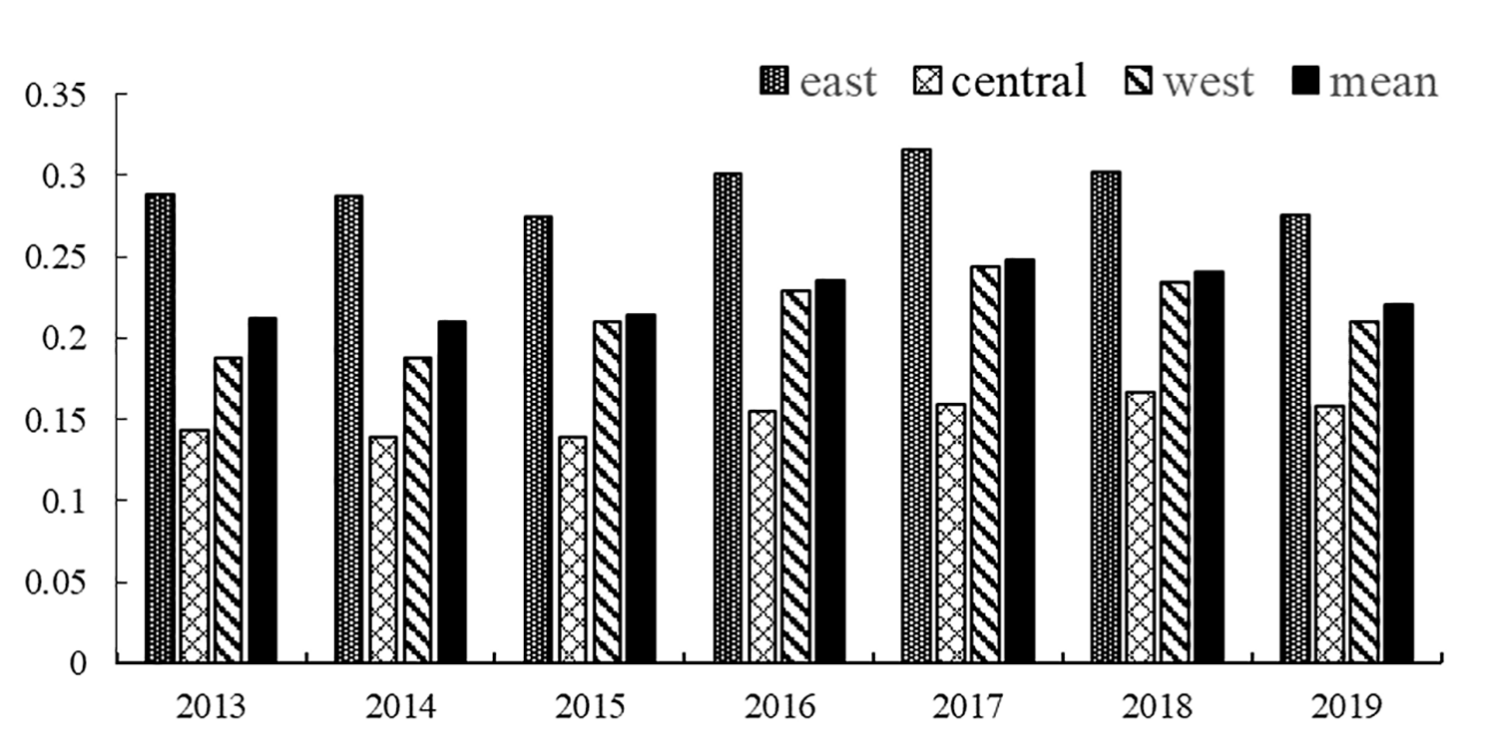
The trend of the HQD of undertakings for the aged in eastern, central and western regions of China from 2013 to 2019

It can be seen from Table 1 that the weights of old-age social security and elderly care service are the largest, 45.61% and 25.46% respectively, indicating they have the largest contribution to the comprehensive evaluation system of the HQD of undertakings for the aged. Figure 1 shows the trend of the HQD of undertakings for the aged in 31 provincial regions of China from 2013 to 2019 (see Table S1 of Additional file 1 for comprehensive index values). It can be seen that the HQD of undertakings for the aged is at relatively low level. The average value of the HQD of China’s undertakings for the aged rose from 0.212 to 2013 to 0.248 in 2017, and then decreased to 0.220 in 2019. The difference between provincial administrative regions was very large, ranging from 0.071 to 0.622. Taking the average of HQD in 2013–2019 as an example, Beijing (0.554) and Shanghai (0.530) were in the leading positions in the HQD of the undertakings for the aged, with distinct advantages over the third (Zhejiang, 0.346), the fourth (Jiangsu, 0.332) and the fifth (Xizang, 0.300). Henan (0.127), Guangxi (0.120), Jilin (0.115) were relatively lagging behind, ranking in the bottom three. Comparing the three regions, we find that the eastern region has the highest annual average of HOD (0.292), followed by the western region (0.215) and the lowest in the central region (0.151). During the period of 2013 to 2019, the coefficient of variation between the three regions rises from 0.481 to 0.498, which indicates the regional gap in the HQD of China’s undertakings for the aged has slightly enlarged.

### Spatial distribution of undertakings for the aged HQD

Table 3 shows the ranges of HQD level classification in 2013–2019 by using the Natural Breaks Classification (Jenks) with the ArcGIS software. It shows that the maximum value of each range had a trend of rising first and then falling from 2013 to 2019, which is consistent with the results of Fig. 1 and Table S1 (See Additional file 1) that the HQD level experienced a process of rising first and then falling.

**Table 3:** Ranges of HQD level classification

Year	Low level area	Medium-low level area	Medium-high level area	High level area
2013	0.082–0.140	0.140–0.205	0.205–0.318	0.318–0.547
2014	0.066–0.123	0.123–0.205	0.205–0.366	0.366–0.584
2015	0.071–0.152	0.152–0.236	0.236–0.391	0.391–0.528
2016	0.114–0.179	0.179–0.256	0.256–0.353	0.353–0.588
2017	0.113–0.132	0.132–0.244	0.244–0.360	0.360–0.622
2018	0.133–0.169	0.169–0.205	0.205–0.301	0.301–0.558
2019	0.105–0.152	0.152–0.197	0.197–0.288	0.288–0.546

**Fig. 2:**
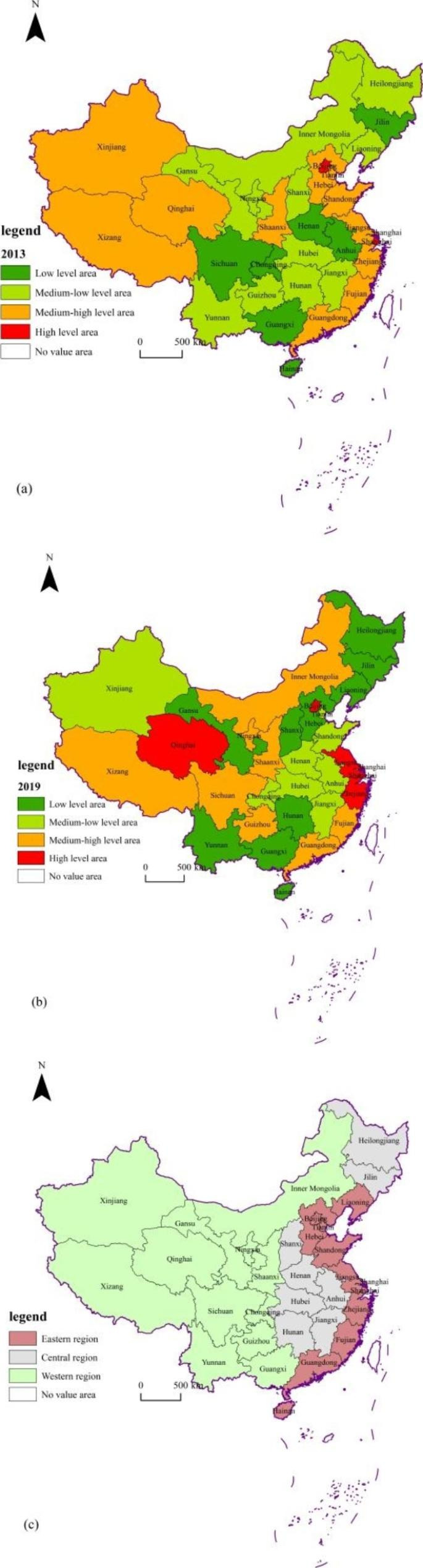
Spatial differences of HQD comprehensive index of China’s undertakings for the aged in 2013 and 2019

Figure 2 shows the spatial distribution of HQD of China’s undertakings for the aged in 2013 (See Fig. 2, Subfigure (a)) and 2019 (See Fig. 2, Subfigure (b)). Subfigure (c) in Fig. 2 indicates the location of China’s eastern, central, and western regions. In 2013, more than 60% provincial administrative regions were at low or medium-low level of HQD; only two regions (Beijing and Shanghai) were at high level; both high-level regions were distributed in the east area. The medium-high and high-level regions were mainly distributed in the eastern and western area. The HQD of undertakings for the aged had shown a core-periphery structure; the core was the two high-level regions; the medium high-level regions were located around the core.

In 2019, the number of high-level provinces increased to five. The proportion of low and medium low-level regions in 2019 was 59%, close to 2013. A dual-core spatial pattern was formed in 2019, where Qinghai (located in western China) and Shanghai (located in eastern China) were the two cores. Similar to 2013, the HQD of the undertakings for the aged in 2019 showed a “high-low-high” trend from east to west, forming a V-shaped pattern of “east-middle-west”.

### Spatial autocorrelation test results

Table 4 shows the results of the spatial autocorrelation test. Except that Moran’s I values in 2015 and 2017 are less than 0.1, they are significantly positive in other years at the 0.05 test level, which indicates that the HQD of the undertakings for the aged has a significant positive spatial correlation. Therefore, it is appropriate to use a spatial econometric model to empirically analyze the spatial effects.

**Table 4:** Moran’s I index of the HQD of undertakings for the aged (2013–2019)

Year	2013	2014	2015	2016	2017	2018	2019
Global Moran’s value	0.286	0.239	0.142	0.191	0.13	0.217	0.173
P-value	0.005***	0.027**	0.073*	0.039**	0.070*	0.024**	0.049**

Note: *, **, and *** indicate significance at the level of 0.1, 0.05 and 0.01 respectively

Table 5 shows cluster types of HQD comprehensive index of various provinces in 2013, 2016 and 2019. The high-high cluster type was mainly distributed in the eastern region; the low-low cluster type was mainly distributed in the western and central regions. Please see the Additional file 2 (Figure s1) for Moran’s scatter plot. The first quadrant represents the high-high cluster area, the second quadrant represents the low-high cluster area, the third quadrant represents the low-low cluster area, and the fourth quadrant represents the high-low cluster area. It can be seen that most provincial administrative regions are in the low-low cluster area.

**Table 5:** Clustering types of HQD comprehensive index of undertakings for the aged in various provincial administrative regions in China (2013–2019)

Year	Type	Provincial administrative regions	Region* (the number of provinces)
2013	High-high	Beijing, Fujian, Hebei, Jiangsu, Shanghai, Tianjin, Zhejiang, Xinjiang	Eastern region (7) Western region (1)
	High-low	Guangdong, Shandong, Qinghai, Shaanxi, Xizang	Eastern region (2) Western region (3)
	Low-high	None	None
	Low-low	Hainan, Liaoning, Chongqing, Gansu, Guangxi, Guizhou, Inner Mongolia, Ningxia, Sichuan, Yunnan, Anhui, Heilongjiang, Henan, Hubei, Hunan, Jiangxi, Jilin, Shanxi	Eastern region (2) Western region (8) Central region (8)
2016	High-high	Fujian, Jiangsu, Shanghai, Zhejiang, Qinghai, Xinjiang, Xizang	Eastern region (4) Western region (3)
	High-low	Beijing, Guangdong, Guizhou, Sichuan	Eastern region (2) Western region (2)
	Low-high	Hebei, Tianjin, Gansu, Yunnan	Eastern region (2) Western region (2)
	Low-low	Hainan, Liaoning, Shandong, Chongqing, Guangxi, Inner Mongolia, Ningxia, Shaanxi, Anhui, Heilongjiang, Henan, Hubei, Hunan, Jiangxi, Jilin, Shanxi	Eastern region (3) Western region (5) Central region (8)
2019	High-high	Fujian, Jiangsu, Shanghai, Zhejiang, Xizang	Eastern region (4) Western region (1)
	High-low	Beijing, Guangdong, Ningxia, Qinghai	Eastern region (2) Western region (2)
	Low-high	Hebei, Tianjin, Gansu, Xinjiang, Anhui, Jiangxi	Eastern region (2) Western region (2) Central region (2)
	Low-low	Hainan, Liaoning, Shandong, Chongqing, Guangxi, Guizhou, Inner Mongolia, Shaanxi, Sichuan, Yunnan, Heilongjiang, Henan, Hubei, Hunan, Jilin, Shanxi	Eastern region (3) Western region (7) Central region (6)

Note: *According to the division of the three major economic zones by the National Bureau of Statistics of China, China is divided into eastern, central and western regions. 11 provinces are in the eastern regions, including Beijing, Tianjin, Hebei, Liaoning, Shanghai, Jiangsu, Zhejiang, Fujian, Shandong, Guangdong and Hainan; 8 provinces are in the central region, including Shanxi, Jilin, Heilongjiang, Anhui, Jiangxi, Henan, Hubei and Hunan; 12 provinces are in the western region, including Tibet, Inner Mongolia, Guangxi, Chongqing, Sichuan, Guizhou, Yunnan, Shaanxi, Gansu, Qinghai, Ningxia and Xinjiang

Table 6 shows the test results related to model selection among SEM, SAR and SDM. Both robust LM-error test and robust LM-lag test are significant, indicating that both SEM and SAR are appropriate. The LR test and Wald test reject the null hypothesis that SDM can be simplified to SEM or SAR, which indicates that SDM is better than SEM and SAR. In order to further compare the SDM with SAC, the statistics including R-squared, AIC, and BIC are showed in Table 7. It can be seen that that the SAC model using binary adjacency matrix (SAC1) having a relatively high R-squared and low values of AIC and BIC, which indicates it is better than SDM and the SAC model based on the geographical distance matrix (SAC2). The Hausman’s test rejects the null hypothesis at 5% significance level, so a fixed effect model is selected.

**Table 6:** The test results related to model selection

		Statistics	P-value
LM test	SEM	19.118 **	0.000
	Robust SEM	25.912 ***	0.000
	SAR	1.320	0.251
	Robust SAR	8.115 **	0.004
LR test	SDM can be simplified to SAR	32.760 ***	0.000
	SDM can be simplified to SEM	31.680 ***	0.000
Wald test	SDM can be simplified to SAR	31.020 ***	0.000
	SDM can be simplified to SEM	23.600 ***	0.000
Hausman test	SAR	15.330 *	0.032
	SEM	16.480 *	0.021
	SDM	38.850 ***	0.000

Note: *, **, and *** indicate significance at the level of 0.05, 0.01 and 0.001 respectively

**Table 7:** Estimated coefficients of the spatial SEM, SAR, SDM, SAC models

Type of variables	Variables	OLS	SEM	SAR	SDM	SAC1	SAC2
Space lag term	Rho			0.148** (0.072)	0.235 *** (0.058)	0.746 *** (0.086)	0.739 *** (0.153)
	Lambda		0.300 *** (0.061)			-0.702 *** (0.173)	-0.487 (0.567)
Economy factors	The GDP per capita	0.034 ** (0.016)	0.038 ** (0.012)	0.033 ** (0.014)	0.049 ** (0.021)	0.036 *** (0.013)	0.034 ** (0.014)
	The proportion of the tertiary industry	0.023 *** (0.004)	0.023 *** (0.005)	0.022 *** (0.005)	0.020 *** (0.006)	0.018 *** (0.005)	0.022 *** (0.006)
Population aging factors	The dependency ratio of the older adults	-0.003 (0.012)	0.001 (0.013)	0.002 (0.014)	-0.004 (0.012)	0.006 (0.010)	-0.002 (0.013)
	The proportion of older adults	-0.074 *** (0.014)	-0.091 *** (0.012)	-0.078 *** (0.013)	-0.098 *** (0.011)	-0.081 *** (0.009)	-0.079 *** (0.012)
The digital technology factors	Ln(the fixed asset investment in digital industries)	0.040 * (0.023)	0.037 ** (0.018)	0.035 * (0.019)	0.027 (0.017)	0.028 * (0.016)	0.036 * (0.018)
	Ln(the number of employees in digital industries)	-0.089 (0.047)	-0.039 (0.093)	0.086 (0.105)	-0.046 (0.103)	0.071 (0.075)	-0.046 (0.095)
R-square		0.309	0.375	0.292	0.201	0.462	0.3465
AIC		-246.339	-254.533	-247.704	-267.524	-267.159	-259.985
BIC		-222.680	-227.494	-220.664	-220.2055	-236.740	-229.566
Log-likelihood		130.169	135.267	131.852	147.762	142.579	138.993

Note: *, ** and *** indicate significance at the level of 0.1, 0.05, 0.01, respectively. Rho, Spatial Autoregressive Parameters; Lambda, Residual lag parameter; Ln, natural logarithm; the SAC1 model is based on binary adjacency matrix, the SAC2 model is based on the geographical distance matrix. The value in parentheses represents the standard deviation

### Spatial panel model results

Table 7 shows the results of the ordinary panel model (OLS) and five spatial panel models of the HQD of the undertakings for the aged in Chinese provincial regions. It can be seen that in the SAC1 model, the spatial autoregressive coefficient is 0.746 and has passed the significance test of 0.01, which indicates that the HQD of the undertakings for the aged in adjacent areas has a significant positive impact on the observed regions. The spatial autoregressive coefficients in SEM, SAR, SDM and SAC2 models are positive, which further indicates that spatial correlation is robust. The coefficient of residual lag term in the SAC1 model is -0.702, which also passes the significance test of 0.01.

According to the results of the SAC1 model in Table 7, there is a significant positive correlation between per capita GDP (coefficient = 0.036, P-value = 0.006), the proportion of the tertiary industry (coefficient = 0.018, P-value = 0.000) and the fixed asset investment in digital industries (coefficient = 0.028, P-value = 0.084). The results of SEM and SAR models are consistent with those of SAC model. The higher the economic level and digital technology, the higher the quality development level of the undertakings for the aged, which means that the development of local economy and digital technology can significantly promote the HQD of local undertakings for the aged. The proportion of the older adults over 60 years old (coefficient=-0.081, P-value = 0.000) has a significantly negative correlation with the HQD of the undertakings for the aged, which indicates that a higher degree of aging will pull down the HQD level of the undertakings for the aged in the region.Therefore, the results of the spatial panel models validated hypotheses 1–3.

## Discussion

This study explored the scientific connotation of the HQD of undertakings for the aged, and established a comprehensive evaluation index system to measure the level of HQD. Based on the panel data from 2013 to 2019, we identified the spatial pattern and driving factors of the HQD of undertakings for the aged in China. The findings of this study help to understand the overall distribution characteristics, spatial effects, and main driving forces of the development of China’s current undertakings for the aged.

### Evolution characteristics of China’s HQD of undertakings for the aged

The study revealed that the HQD level of undertakings for the aged is slowly rising. The undertakings for the aged are essentially the behavior of the government in social public management, and its development depends on the government’s planning and policy support [4]. Since the Chinese government has implemented policies to promote the HQD of undertakings for the aged, the slow upward trend of the overall development level of undertakings for the aged may be related to the implementation of the policies [6].

However, the HQD of China’s undertakings for the aged is still at low level. The value of the comprehensive level of the HQD was in the lowest quartile. About 60% of provincial administrative regions were at a low or medium-low level of HQD during the period from 2013 to 2016. According to reference [31], the HQD of China’s undertakings for the aged could be considered at a low level when the proportion of low and medium-low levels is relatively high. One possible reason is that the central government’s financial expenditure on the elderly is insufficient. The fiscal weight of China’s social programs is still light by international comparison. In 2020, China’s fiscal expenditure on social security accounted for only 3.2% of GDP, far lower than 18% in the United States and 22% in Japan. Besides, the production field is more favored by local governments, while the fiscal expenditure in welfare and security is much lower [52]. Another possible reason is that the expansion of the market economy has not effectively promoted the socialization of elder care services. The construction of China’s socialized elder care service system is a government-led model with the participation of a variety of social forces [53]. In practice, the elder care service resources are still limited to the government as a single source supplier. Large initial investment, long payback period, uncertain income and high barriers to entry lead to insufficient participation of social forces [54]. The insufficient and unbalanced development of socialized elder care services has hindered the HQD of China’s undertakings for the aged.

The findings in this study show large spatial differences in the HQD of China’s undertakings for the aged. The level of HQD of the undertakings for the aged in the economically developed eastern regions and the policy-supported western regions is higher than that in the central regions. This finding is consistent with the results of existing literature [31], although it establishes an index system different from this study to evaluate the development level of undertakings for the aged. The level of HQD of the undertakings for the aged in the eastern region is the highest. The possible reason is that the eastern region has the most developed economy, which makes it possible to provide strong financial support for the undertakings for the aged. In addition, the service industry in the eastern region is the most advanced and it benefits the provision of the elder service. The relatively high level of HQD of the undertakings for the aged in western region may be explained in two aspects, the support from the nation’s old-age industry policy and the low level of population aging in the western region. From the perspective of spatial evolution trend, there was only one center clustered in the eastern region in 2013, and two centers clustered in the eastern and western regions in 2019, which reflects the high-quality development of the undertakings for the aged in the western region. Therefore, it’s important to improve the HQD of the undertakings for the aged for the lagging-behind central area in order to achieve balanced development, which is vital for the overall HQD of undertakings for the aged.

### Spatial effects of the HQD of undertakings for the aged

This study revealed that there was a significant spatial effect on the HQD of the undertakings for the aged. The local HQD of the undertakings for the aged is affected by neighboring regions. First, the undertakings for the aged are public goods with low geographical restrictions. If there are enough elder care facilities in adjacent areas to meet the local demand, local governments will be less motivated to spend on it, which is called substitution effect generated by spillover effect. The provision of elder service is included in the performance appraisal of some local governments. However, in the assessment system that emphasize on GDP growth, the local government officials pay less attention to the fields of people’s livelihood needs than those which can take obvious achievements in a short term [55]. This leads to insufficient motivation for the development of undertakings for the aged. Second, local governments with better performance receive more support from the central government, and the officials have better opportunities for promotion. Thus, on the one hand, local governments increase the overall supply of public goods to catch up with the level of public services in adjacent areas, which is called catch-up effect. On the other hand, local governments might increase the supply of productive public goods to attract capital and highly skilled workforce. We call this competitive effect. These two effects make the spatial interaction an important factor in the analysis of local HQD of undertakings for the aged.

### Policy implications

#### Developing the monitoring system of the HQD of undertakings for the aged

As a strategic action to actively cope with the aging of the population, there is no doubt that the HQD of undertakings for the aged will contribute to the sustainability of social policy systems for the older adults. But this acknowledgment is not enough to ensure that it is well implemented in practice. Therefore, it is necessary to form a unified understanding and make evaluations on the HQD of undertakings for the aged so that the gaps in development could be found out and targeted policies could be formulated.

This study constructed a four-dimension indicator system of HQD of undertakings for the aged to evaluate the change in the development level and to identify the lagging areas in a certain dimension. The HQD indicator system of undertakings for the aged makes it possible to dynamically monitor the evolution trend and spatial pattern of HQD, and to find out the gaps in development. The inclusion of the indicator system into the development plan of undertakings for the aged and the formation of the development goals are conducive to guiding the undertakings for the aged towards the goal of high-quality development.

#### Strategies for promoting the HQD of undertakings for the aged

In order to put forward some targeted policies to continuously improve and strengthen the level of HQD of undertakings for the aged, we propose to focus on improving the incentive mechanism in different regions to guide the local government to establish multi-dimensional comprehensive goals, including maintaining sustainable economic development, developing digital technology and strengthening policy top-level design.

This empirical study shows that economic development has a significant positive impact on the HQD of the undertakings for the aged. As a comprehensive social construction and public service project, the construction and development of the undertakings for the aged cannot be separated from economic support. Economic development is the primary driving force to promote the construction of the old-age security system and elder care service system [36]. Long-term economic growth enhances the government’s ability to provide subsidies and insurance benefits for the older adults by providing strong financial resources for local governments to carry out ageing-related activities [56]. In addition, the improvement of the level of economic development often results in the increase of residents’ income, as well as the improvement of their willingness and ability to pay for elder care service [57]. The prosperity of the old-age consumption and service market helps to promote the HQD of undertakings for the aged.

This study provides evidence that digital technology has a significant positive impact on the HQD of undertakings for the aged. Digital technology has become a significant influencing factor of high-quality economic development [17]. Digital governance is worthy of attention. We might reshape the concept of elder care services and improve the HQD of undertakings for the aged by promoting digitization and intelligence. It is suggested to explore a new model of elderly care in the era of “digital intelligence”. Intelligent technologies that effectively connect families, communities, governments, medical institutions and social organizations could be used to build a service network for the older adults so that the social participation of the older adults could be improved.

In addition, the government’s planning and policy support play a role in the development of the undertakings for the aged. First, the government should be committed to establishing a fair and inclusive basic old-age security system, basic medical insurance system and long-term care insurance system. Overall plans could be made to improve the systems of social assistance, social welfare, philanthropy, preferential treatment and resettlement. Since China’s long-term care insurance system is still at the pilot stage, it is urgent to build a national long-term care insurance system as soon as possible to provide long-term care security for the elderly people with disability. Second, in order to improve the elderly care service system, the government could establish an incentive mechanism to encourage the provision of affordable socialized high-quality elderly care services. For instance, local governments provide financial subsidies or tax incentives to small start-up elderly care facilities. Third, it is necessary to strengthen the development of undertakings for the aged while adhering to the principle of balanced development. Considering that the evaluation dimension of old-age social security has the highest weight (Table 1), promoting balanced economic development could help to reduce the gaps of local economic support for the undertakings for the aged. For the central region where the development of the HQD of the undertakings for the aged was lagging behind, the central government should increase policy support to avoid further widening the gap.

We recognize the limitations of this study. First, the policy factor is not considered in the empirical research. The one reason is that the policy force is not easy to quantify. The second reason is that the policy factor often interacts with economic factors, which can affect the independence of explanatory variables if it is included in the model. The second weak point is small sample size. Only seven years of panel data are used in the study due to the limitation of data availability. In future study, we will try to figure out the solutions to the above problems. Data for a longer period of time would be collected in order to find a more convincing law of development of the undertakings for the aged.

## Conclusion

This study measures the level of HQD of China’s undertakings for the aged and analyzes the evolution of spatial pattern and the driving factors of HQD of undertakings for the aged. The main conclusions are as follows: First, the HQD of China’s undertakings for the aged was rising volatility, but the overall level was low. Second, the HQD of the undertakings for the aged showed significant spatial differences, forming a “high-low-high” trend from eastern to western China. The aggregation type evolved from single-core to dual-core during the period of 2013–2019. Third, economic development and digital technology had positive impact on the HQD of undertakings for the aged, while population aging had a negative impact. It is suggested that the governments should enhance the evaluation system of the HQD of undertakings for the aged to find out development gaps, continuously improve economic development and technological progress to promote the HQD of undertakings for the aged. Overall, this study provides a theoretical basis and data-based evidences for the government to formulate policies for the high-quality development of undertakings for the aged.

## Electronic Supplementary Material

Below is the link to the electronic supplementary material.

Supplementary Material 1

Supplementary Material 2

## Data Availability

Publicly available datasets were analyzed in this study. This data can be found here: http://www.stats.gov.cn/tjsj/ndsj/, https://www.mca.gov.cn/article/sj/, https://www.yearbookchina.com/navibooklist-n3020013350-1.html. The datasets used and/or analyzed during the current study are also available from the corresponding author on reasonable request.
