# Supplementary material for: Spatial differentiation and driving factors of the high-quality development of undertakings for the aged of China
Source: Int J Equity Health. 2023 May 26;22:104. doi: 10.1186/s12939-023-01921-7 (PMC10214618; doi:10.1186/s12939-023-01921-7)
Supplement: Supplementary file 1 — Supplementary Material 1 [file 12939_2023_1921_MOESM1_ESM.docx]

Additional file 1

Table S1 The HQD comprehensive index of China's undertakings for the aged (2013-2019)

| Provinces | 2013 | 2014 | 2015 | 2016 | 2017 | 2018 | 2019 | Mean | Ranking |
| --- | --- | --- | --- | --- | --- | --- | --- | --- | --- |
| Beijing | 0.547 | 0.510 | 0.508 | 0.588 | 0.622 | 0.558 | 0.546 | 0.554 | 1 |
| Shanghai | 0.512 | 0.584 | 0.528 | 0.565 | 0.503 | 0.515 | 0.501 | 0.530 | 2 |
| Zhejiang | 0.318 | 0.366 | 0.312 | 0.345 | 0.360 | 0.373 | 0.350 | 0.346 | 3 |
| Jiangsu | 0.270 | 0.299 | 0.304 | 0.343 | 0.347 | 0.383 | 0.375 | 0.332 | 4 |
| Xizang | 0.274 | 0.263 | 0.390 | 0.353 | 0.354 | 0.241 | 0.224 | 0.300 | 5 |
| Qinghai | 0.254 | 0.218 | 0.222 | 0.269 | 0.293 | 0.399 | 0.426 | 0.297 | 6 |
| Guizhou | 0.205 | 0.294 | 0.375 | 0.304 | 0.321 | 0.267 | 0.208 | 0.282 | 7 |
| Fujian | 0.229 | 0.229 | 0.272 | 0.301 | 0.323 | 0.300 | 0.288 | 0.277 | 8 |
| Guangdong | 0.233 | 0.255 | 0.236 | 0.256 | 0.309 | 0.301 | 0.253 | 0.263 | 9 |
| Xinjiang | 0.215 | 0.234 | 0.235 | 0.271 | 0.282 | 0.275 | 0.194 | 0.244 | 10 |
| Tianjin | 0.262 | 0.227 | 0.202 | 0.225 | 0.244 | 0.235 | 0.189 | 0.226 | 11 |
| Sichuan | 0.129 | 0.193 | 0.210 | 0.303 | 0.283 | 0.253 | 0.212 | 0.226 | 12 |
| Ningxia | 0.192 | 0.200 | 0.188 | 0.212 | 0.298 | 0.249 | 0.221 | 0.223 | 13 |
| Shaanxi | 0.218 | 0.205 | 0.209 | 0.222 | 0.258 | 0.226 | 0.211 | 0.221 | 14 |
| Shandong | 0.261 | 0.237 | 0.218 | 0.226 | 0.267 | 0.169 | 0.162 | 0.220 | 15 |
| Inner Mongolia | 0.176 | 0.154 | 0.172 | 0.201 | 0.227 | 0.228 | 0.216 | 0.196 | 16 |
| Shanxi | 0.196 | 0.205 | 0.176 | 0.206 | 0.198 | 0.160 | 0.139 | 0.183 | 17 |
| Hubei | 0.151 | 0.158 | 0.167 | 0.197 | 0.180 | 0.205 | 0.197 | 0.179 | 18 |
| Hebei | 0.243 | 0.151 | 0.179 | 0.159 | 0.167 | 0.163 | 0.139 | 0.172 | 19 |
| Yunnan | 0.175 | 0.162 | 0.166 | 0.179 | 0.193 | 0.155 | 0.152 | 0.169 | 20 |
| Liaoning | 0.176 | 0.178 | 0.152 | 0.163 | 0.169 | 0.158 | 0.125 | 0.160 | 21 |
| Heilongjiang | 0.152 | 0.123 | 0.199 | 0.173 | 0.113 | 0.191 | 0.136 | 0.155 | 22 |
| Anhui | 0.140 | 0.143 | 0.129 | 0.141 | 0.182 | 0.178 | 0.161 | 0.153 | 23 |
| Hunan | 0.162 | 0.136 | 0.134 | 0.140 | 0.181 | 0.163 | 0.144 | 0.151 | 24 |
| Gansu | 0.158 | 0.146 | 0.143 | 0.156 | 0.132 | 0.177 | 0.146 | 0.151 | 25 |
| Jiangxi | 0.156 | 0.140 | 0.121 | 0.153 | 0.131 | 0.158 | 0.172 | 0.147 | 26 |
| Chongqing | 0.132 | 0.114 | 0.118 | 0.157 | 0.165 | 0.181 | 0.164 | 0.147 | 27 |
| Hainan | 0.120 | 0.120 | 0.104 | 0.135 | 0.161 | 0.167 | 0.105 | 0.130 | 28 |
| Henan | 0.107 | 0.120 | 0.119 | 0.114 | 0.122 | 0.133 | 0.177 | 0.127 | 29 |
| Guangxi | 0.125 | 0.066 | 0.097 | 0.123 | 0.123 | 0.156 | 0.147 | 0.120 | 30 |
| Jilin | 0.082 | 0.083 | 0.071 | 0.118 | 0.164 | 0.143 | 0.141 | 0.115 | 31 |
| Eastern Region* | 0.288 | 0.287 | 0.274 | 0.301 | 0.316 | 0.302 | 0.276 | 0.292 | 1 |
| Central Region* | 0.143 | 0.139 | 0.139 | 0.155 | 0.159 | 0.166 | 0.159 | 0.151 | 3 |
| Western Region* | 0.188 | 0.187 | 0.210 | 0.229 | 0.244 | 0.234 | 0.210 | 0.215 | 2 |
| Mean | 0.212 | 0.210 | 0.215 | 0.235 | 0.248 | 0.241 | 0.220 | 0.226 | - |
| Coefficient of variation | 0.481 | 0.534 | 0.517 | 0.490 | 0.461 | 0.444 | 0.498 | 0.469 | - |

*According to the division of the three major economic zones by the National Bureau of Statistics of China, China is divided into eastern, central and western regions. 11 provinces are in the eastern regions, including Beijing, Tianjin, Hebei, Liaoning, Shanghai, Jiangsu, Zhejiang, Fujian, Shandong, Guangdong and Hainan; 8 provinces are in the central region, including Shanxi, Jilin, Heilongjiang, Anhui, Jiangxi, Henan, Hubei and Hunan; 12 provinces are in the western region, including Tibet, Inner Mongolia, Guangxi, Chongqing, Sichuan, Guizhou, Yunnan, Shaanxi, Gansu, Qinghai, Ningxia and Xinjiang.
