# Supplementary figures and images for: Spatial differentiation and driving factors of the high-quality development of undertakings for the aged of China
Source: Int J Equity Health. 2023 May 26;22:104. doi: 10.1186/s12939-023-01921-7 (PMC10214618; doi:10.1186/s12939-023-01921-7)

Additional file 2

Figure s1 Moran’s scatter plot (2013-2019)


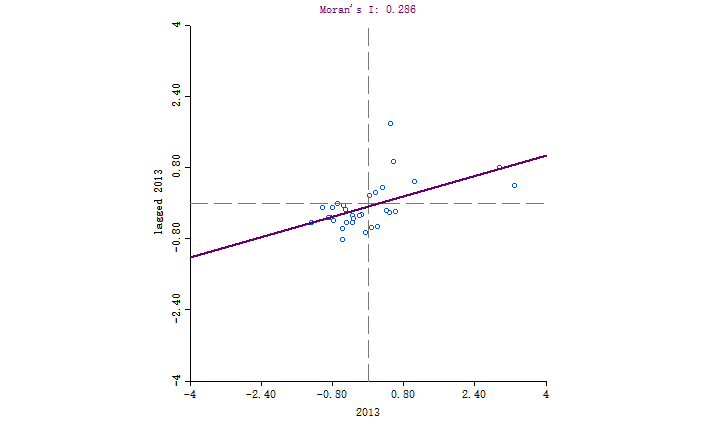

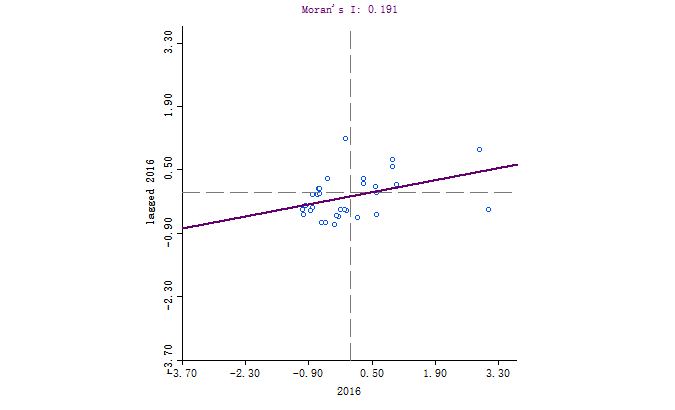

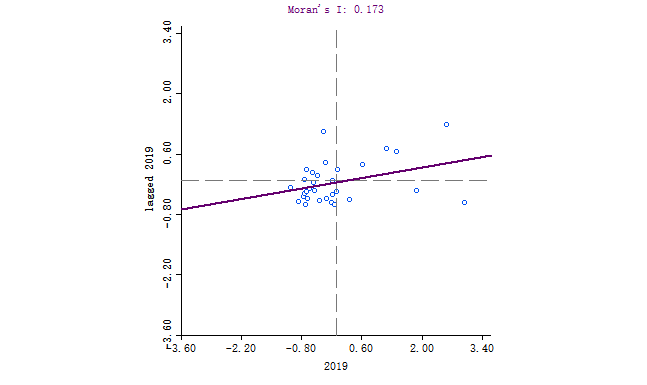

Supplement: Supplementary file 2 — Supplementary Material 2 [file 12939_2023_1921_MOESM2_ESM.docx]
